# Artificial intelligence in shoulder and elbow surgery: a bibliometric analysis of affiliation-based collaboration patterns

**DOI:** 10.1016/j.jseint.2025.08.017

**Published:** 2025-10-14

**Authors:** Ausberto R. Velasquez Garcia, Valeria Vismara, Sergio F. Guarin Perez, Fernando Radice

**Affiliations:** a Mayo Clinic Department of Orthopedic Surgery, Rochester, MN, USA; b Department of Orthopedic Surgery, Clinica Universidad de Los Andes, Santiago, Chile; c Department of Orthopedic Surgery, Università Degli Studi di Milano, Milano, Italy; d School of Medicine, Universidad de los Andes, Santiago, Chile

**Keywords:** Artificial intelligence, Machine learning, Shoulder surgery, Elbow surgery, Affiliation-based collaboration, Bibliometric analysis

## Abstract

**Background:**

Despite growing interest, artificial intelligence (AI) applications in shoulder and elbow surgery remain underdeveloped. While adoption is accelerating and shows promise in addressing complex clinical problems, substantial technical and clinical barriers persist. Collaborative research may be relevant for generating high-quality datasets and more robust, generalizable, and clinically relevant algorithms. This study aimed to 1) analyze trends in AI research productivity and impact, 2) map collaboration patterns among affiliations and regions, and 3) assess the relationship between affiliation-based collaboration and research outcomes.

**Methods:**

We conducted a bibliometric analysis of Scopus-indexed articles published between January 2000 and November 2024, focusing on peer-reviewed studies involving AI applications in shoulder or elbow surgery. Data collected included number of publications, citation metrics, author affiliations, and index keywords. These variables were used to calculate composite metrics and to examine the geographic distribution of research and collaboration patterns using network analysis. Two linear regression models assessed the relationship between affiliation-based collaborations and publication volume and citation impact.

**Results:**

Of 181 identified scholarly documents, 119 met eligibility criteria. These articles were published across 63 journals and cited a total of 1,519 times. The *Journal of Shoulder and Elbow Surgery* contributed the highest number of articles (n = 20), while *Acta Orthopaedica* had the highest average citations per article (n = 310), although in a single article. The annual publication rate increased rapidly after 2014, peaking at 49 in 2024. A small group of affiliations disproportionately influenced output and citations. Collaboration networks were sparse (density 0.03) yet showed distinct geographic clusters. Most research output originated from the United States (48%), followed by South Korea (12%) and China (8%). A total of 1,010 collaborations were identified among 260 affiliations. The network showed low density (0.03) but high modularity (0.81), indicating sparse overall connectivity yet tightly clustered communities. Regression models indicated that each additional collaboration established between affiliations was associated with 5 more publications (R^2^ = 1.0) and increased average citations per article by 0.2 (R^2^ = 0.77).

**Conclusion:**

Affiliation-based collaboration was strongly associated with both the volume and citation impact of AI research in shoulder and elbow surgery. Strengthening and expanding these networks may enhance global research participation, foster innovation, and improve the clinical applicability of future work.

Artificial intelligence (AI) is at the forefront of transformative developments in orthopedic surgery, promising substantial advancements in diagnostic accuracy, surgical planning, and outcome prediction.^18^^,^^25^^,^^43^^,^^47^^,^^59^^,^^62^ In shoulder and elbow surgery, AI applications are rapidly increasing and have demonstrated the potential to address complex clinical challenges ranging from enhanced precision in imaging-based diagnostics and risk prediction models to surgical outcome prediction.^24^^,^^29^^,^^33^^,^^34^^,^^36^^,^^44^^,^^60^

Despite growing interest, AI research in shoulder and elbow surgery remains underdeveloped, facing several technical and clinical challenges.^23^^,^^28^^,^^29^^,^^40^^,^^45^^,^^52^^,^^62^ Although AI has the potential to enhance decision-making processes, substantial progress is needed before it can be routinely implemented into clinical practice.^4^^,^^5^^,^^17^^,^^56^ Current AI models lack robust performance or have limited external validation, highlighting the need for greater scientific rigor.^19^^,^^27^^,^^36^ Many algorithms are often trained on small and relatively homogenous datasets, which may limit their generalizability to diverse patient populations and settings.^23^^,^^49^ Additionally, the absence of standardized validation protocols and reproducibility further undermines the reliability and clinical utility of these models.^61^

It is institutive that collaborative efforts could derive in more extensive datasets and potentially more robust algorithms that can enhance accuracy, generalizability, and clinical relevance.^23^ Cross-affiliation collaborations can be fundamental for addressing data scarcity and algorithm validation challenges, which are barriers to AI's broader clinical integration.^36^^,^^66^ Such collaborations drive the creation of innovative, clinically applicable AI solutions, potentially benefitting patient care in shoulder and elbow surgery. Understanding the current geographic distribution of research is relevant for assessing regional disparities in technological development and adoption, which are potentially influenced by economic and infrastructural variations.^50^^,^^58^ This assessment can be helpful for formulating strategies to elevate global research capabilities, aiming for widespread patient benefits from AI advancements.^58^

Bibliometric analyses are proven approaches in various medical fields for evaluating research activity and trends.^15^^,^^55^ Such analyses could reveal the forces that shape research productivity and impact, including the influence of organizational leadership, geographic distribution, and interdisciplinary collaboration.^55^ This study aimed to 1) analyze trends in AI research productivity and impact in shoulder and elbow surgery, 2) map collaboration patterns among affiliations and regions, and 3) assess the relationship between affiliation-based collaboration and research outcomes. We hypothesized that a small subset of organizations would contribute disproportionately to the research output of the field. Additionally, strong affiliation-based collaboration is expected to correlate positively with higher publication productivity and impact.

## Material and methods

### Data source and search strategy

We used the Scopus database, based on its comprehensive indexing of peer-reviewed literature and robust citation analysis tools. This database provides extensive coverage of journals, conference proceedings, and other academic publications relevant to AI and orthopedic research, ensuring accessibility and completeness for this study. A systematic search was conducted in November 2024 to identify the relevant academic documents focusing on AI applications in shoulder or elbow surgery (Supplemental File 1). Affiliation was defined as the main institutional or organizational entity listed by each author at the time of publication. Collaboration was defined as coauthorship between researchers from different affiliations, serving as a practical and reproducible proxy for cross-institutional collaboration. These definitions were applied consistently across all analyses.

### Inclusion and exclusion criteria

Peer-reviewed journal articles, review articles, and conference proceedings were included to capture a wide range of scholarly contributions. Conference proceedings were included as they can present emerging trends and innovative methodologies. Papers were selected regardless of their original language if they had an English abstract for accessibility in the bibliometric analysis. Documents from January 2000 to November 2024 with complete metadata, including titles, abstracts, keywords, author information, affiliations, and citation counts, were eligible.

Editorials, commentaries, letters to the editor, and other nonresearch articles were excluded. Articles that did not directly address the application of AI in shoulder or elbow surgery or those that focused simultaneously on other orthopedic areas without relevance to the subspecialty were excluded. Preprint and standalone conference abstracts were excluded because of their preliminary nature and lack of formal peer review.

### Article selection and metadata preprocessing

The study selection process consisted of 3 stages: reviewing titles and abstracts to remove irrelevant and duplicate entries, assessing articles for inclusion criteria and relevance, and including eligible articles in the final dataset. Two reviewers (A.V.G., S.G.P.) independently conducted the screening and selection processes to ensure accuracy and resolve discrepancies through consensus or arbitration by the senior author when necessary. From articles that met eligibility criteria, affiliations were standardized to ensure consistency in the analysis, addressing variations in names, abbreviations, spellings, and formatting. Automated scripts were used to perform initial harmonization, followed by manual verification.

### Bibliometric variables

To assess research productivity and impact, we extracted a set of bibliometric metrics from the included dataset. The number of publications (NP) was calculated within the total number of eligible articles, including the overall number or the number of studies according to affiliations, journals, or authors. We also analyzed publications per year to describe trends in scholarly output over time (Table I).

**Table I:** Summary of metrics for research productivity, collaboration, and thematic analysis

Metric	Definition	Interpretation
**Research productivity and impact metrics**		
Number of publications (NP)	Total number of eligible articles included in the dataset	Overall research productivity in the field
Publications per year (PPY)	Number of included articles published each year	Temporal trend in publication activity
Total citation count (TCC)	Sum of citations received by all articles from a journal or author	Cumulative scholarly impact of journals or authors
Average citations (AC)	Total citations divided by number of articles for a journal or institution	Normalized citation impact at the journal or institution level
Citation contribution percentage (CCP)	Percentage of total citations attributed to a specific journal or group	Relative influence of a journal or group within the citation landscape
**Affiliation-based collaboration metrics**		
Number of collaborations	Count of unique coauthorships between different affiliations	Level of collaborative activity across institutions
Degree centrality	Number of direct coauthorship links per affiliation in the network	Institutional prominence based on collaborative links
Network density	Proportion of actual to possible coauthorship links in the network	Overall connectedness of the research network
Modularity (Louvain method)	Measure of how strongly the network is divided into separate communities	Degree to which the network forms tightly knit collaborative clusters
**Authorship and keyword metrics**		
Number of contributing authors (NCA)	Total number of distinct authors across all articles in the dataset	Scope of author participation in the field
Collaboration index (CI)	Average number of authors per article	Degree of collaborative authorship per article

To assess journal-level academic impact, we applied 4 citation-based metrics (Table I):

1) Total citation count
2) Average citations (AC) per article
3) Citation contribution percentage
4) NP per journal.

### Affiliation-based collaboration and geographic distribution

For consistency and to simplify analysis, affiliation-level research output was determined based solely on the first author's institution. Similarly, geographic distribution was assessed by assigning each article to the country of the first author's affiliation. As a result, both country-level and affiliation-level research outputs were calculated by aggregating these first-author records. Data were processed in Python (Pandas Development Team, USA), and results were exported to Excel (Microsoft Corp., Redmond, WA, USA) for visualization.

### Affiliation-based collaboration network visualization

To identify leading research hubs in AI in shoulder and elbow surgery, we calculated the number of articles associated with each author's affiliation. Affiliation-based collaboration was qualitatively analyzed using a coauthorship network constructed in VOSviewer (Centre for Science and Technology Studies at Leiden University, Leiden, Netherlands).^16^ In this network, nodes represented affiliations, with node size reflecting the NP and edge thickness corresponding to the frequency and strength of coauthorship links. Clusters were formed based on the density and strength of connections, highlighting the affiliations with closely linked research activities.

### Affiliation-based collaboration network metrics

Four structural properties of the network were calculated to assess the nature of collaboration (Table I):

1. Number of collaborations
2. Degree centrality
3. Network density
4. Modularity.

All network metrics were derived using the full set of coauthor affiliations for each article, not just those of the first author, to accurately capture the structure and extent of cross-affiliation collaboration. We used the NetworkX Python (Python Software Foundation, Wilmington, DE, USA) library to compute the 4 network metrics. Number of collaborations, the total count of coauthorship links between distinct affiliations, reflects the overall volume of partnership activity. Degree centrality counts the direct links connected to each node and indicates how many partners an affiliation has. Network density divides the observed collaborations by all possible collaborations; values near 0 denote a sparse network, whereas values near 1 indicate extensive interconnection. Modularity, calculated with the Louvain method, measures the strength of community structure; higher values reveal tightly knit clusters of institutions with little overlap.^7^

### Regression modeling on collaboration

Regression models were based on network-level metrics derived from all listed author affiliations, ensuring that collaboration was assessed using the complete coauthorship structure. We explored whether institutional collaboration was linked to research output and citation impact. For each affiliation, we measured collaboration using degree centrality, which counts how many other institutions it coauthored with in the network. The Python statsmodels library was used for Ordinary Least Squares regression modeling. Two regression models were developed.

1. Model 1: tested whether institutions with more collaborators produced a higher NP.
2. Model 2: examined whether these institutions also received more citations per article, measured as AC.

We reported the coefficient of determination (R^2^), regression coefficients, and *P* values for each model. Statistical significance was set at *P* < .05.

### Authorship and keyword analysis

To assess collaborative patterns among researchers, we extracted authorship and keyword metrics from the included dataset (Table I). The following variables were used to describe author participation and collaboration structure.

1. Number of contributing authors (NCA): Reflecting the total number of unique authors across all articles
2. Collaboration index: Average number of authors per article, calculated as NCA divided by NP.

Coauthorship networks were also visualized using VOSviewer. To explore research themes, we analyzed index keywords provided by the authors. Keyword co-occurrence was mapped in VOSviewer to identify clusters of related topics and reveal common research areas and emerging trends.

## Results

### Study selection and publication trends

**Figure 1:**
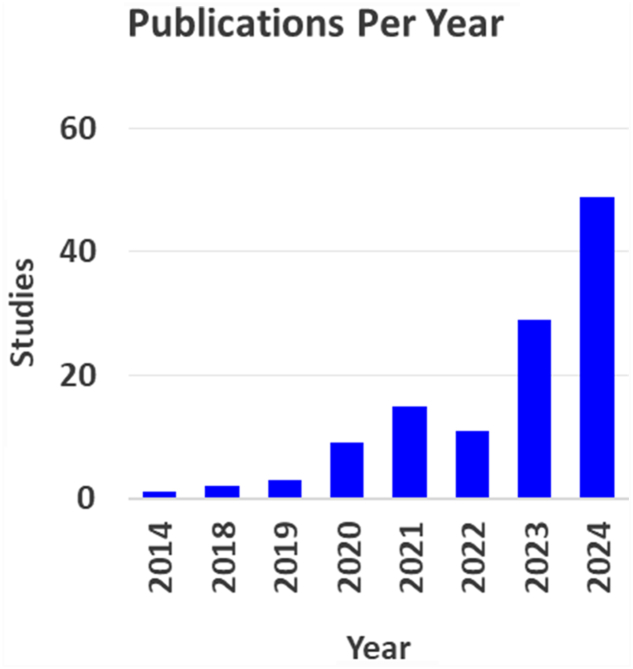
Artificial intelligence studies in shoulder and elbow surgery per year.

A total of 181 studies were identified, of which 119 met the eligibility criteria. These studies were published between 2014 and 2024. No eligible articles were identified between 2000 and 2013. Publication trends demonstrate a consistent upward trajectory in scholarly output over the years, with one publication in 2014 and a steady rise to a peak of 49 publications by 2024. This growth has accelerated notably since 2020, suggesting increasing interest and advancements in the field (Fig. 1).

### Journal contributions and citation metrics

A total of 119 articles were published in 63 journals, with a cumulative citation count of 1,519. The *Journal of Shoulder and Elbow Surgery* (*JSES*) led to 20 publications and 427 citations. *Acta Orthopaedica* achieved 310 citations from a single article (Table I). Emerging journals such as *Seminars in Arthroplasty JSES* (8 publications, 35 citations) and *JSES International* (7 publications, 42 citations) reflect growing specialization in the field. Interdisciplinary platforms, such as *Computational and Structural Biotechnology Journal* (1 publication, 40 citations) and *Radiology: Artificial Intelligence* (1 publication, 17 citations).

The analysis of AC per article (ACPA) and citation contribution percentage indicates that *Acta Orthopaedica* (310 ACPA) and the *Journal of the American Academy of Orthopaedic Surgeons* (63 ACPA) stand out for per-article impact. Meanwhile, *JSES* (33% of total citations) and *Acta Orthopaedica* (24%) dominated the cumulative citation contributions (Table II). The top 10 most-cited articles are listed in Table III.

**Table II:** Top 20 journals with publications on artificial intelligence in shoulder and elbow surgery (ranked by citations)

Source	Studies	Citations	Averagecitations (AC)	Citation contributionpercentage (CCP)
*Journal of Shoulder and Elbow Surgery (JSES)*	20	427	21	28
*Acta Orthopaedica*	1	310	310	20
*The Journal of the American Academy of Orthopaedic Surgeons*	1	63	63	4
*Clinical Orthopaedics and Related Research*	2	59	30	4
*European Radiology*	4	55	14	4
*American Journal of Roentgenology*	1	51	51	3
*Arthroscopy - Journal of Arthroscopic and Related Surgery*	3	51	17	3
*Journal of Personalized Medicine*	2	44	22	3
*JSES International*	7	42	6	3
*Skeletal Radiology*	3	41	14	3
*Computational and Structural Biotechnology Journal*	1	40	40	3
*Seminars in Arthroplasty JSES*	8	35	4	2
*Computer Methods and Programs in Biomedicine*	1	33	33	2
*JBJS Open Access*	1	23	23	2
*Orthopaedic Journal of Sports Medicine*	2	23	12	2
*Sensors*	1	21	21	1
*JSES Reviews, Reports, and Techniques*	2	19	10	1
*Healthcare (Switzerland)*	1	17	17	1
*Radiology: Artificial Intelligence*	1	17	17	1
Coatings	1	14	14	1

**Table III:** Top 10 most-cited articles on artificial intelligence in shoulder and elbow surgery

Title	Author	Year	Source	Cited by
Automated detection and classification of the proximal humerus fracture by using deep learning algorithm^12^	Chung, Seok Won et al.	2018	*Acta Orthopaedica*	310
Construct validation of machine learning in the prediction of short-term postoperative complications following total shoulder arthroplasty^20^	Gowd, Anirudh K.et al.	2019	*Journal of Shoulder and Elbow Surgery*	64
A Novel Machine Learning Model Developed to Assist in Patient Selection for Outpatient Total Shoulder Arthroplasty^6^	Biron, Dustin R. et al.	2020	*The Journal of the American Academy of Orthopaedic Surgeons*	63
Validation of a machine learning–derived clinical metric to quantify outcomes after total shoulder arthroplasty^53^	Roche, Christopher et al.	2021	*Journal of Shoulder and Elbow Surgery*	62
What Is the Accuracy of Three Different Machine Learning Techniques to Predict Clinical Outcomes after Shoulder Arthroplasty?^34^	Kumar, Vikas et al.	2020	*Clinical Orthopaedics and Related Research*	57
Image Quality and Diagnostic Performance of Accelerated Shoulder MRI With Deep Learning-Based Reconstruction^26^	Hahn, Seok et al.	2022	*American Journal of Roentgenology*	51
Using machine learning to predict clinical outcomes after shoulder arthroplasty with a minimal feature set^35^	Kumar, Vikas et al.	2021	*Journal of Shoulder and Elbow Surgery*	49
The value of artificial neural networks for predicting length of stay, discharge disposition, and inpatient costs after anatomic and reverse shoulder arthroplasty^30^	Karnuta, Jaret M. et al.	2020	*Journal of Shoulder and Elbow Surgery*	48
Development of supervised machine learning algorithms for prediction of satisfaction at 2 y following total shoulder arthroplasty^51^	Polce, Evan M. et al.	2021	*Journal of Shoulder and Elbow Surgery*	45
Automated detection and classification of shoulder arthroplasty models using deep learning^65^	Yi, Paul H. et al.	2020	*Skeletal Radiology*	41

### Geographic distribution of research

**Figure 2:**
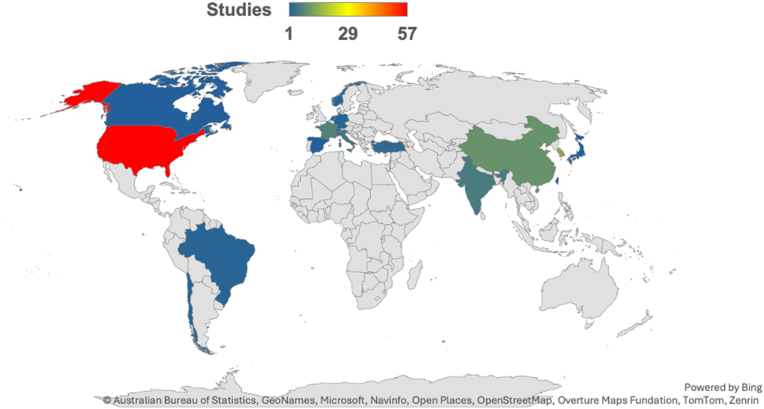
Global distribution of research output of artificial intelligence in shoulder and elbow surgery. Article counts by first author’s affiliation country.

**Figure 3:**
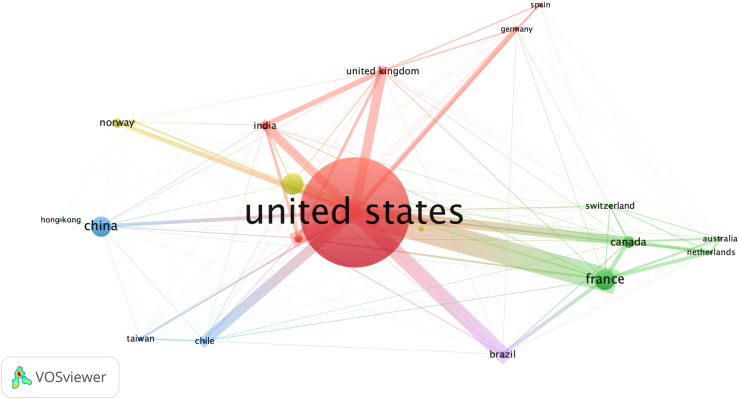
Network visualization of international collaborations in artificial intelligence research for shoulder and elbow surgery.

The analysis of research output by country of first author’s affiliation highlighted a diverse and geographically widespread research landscape (Fig. 2). The United States has 57 articles (48%), followed by South Korea (14 articles, 12%) and China (10 articles, 8%). European countries, including France, 7 publications (6%), 5 publications in Italy (4%), and Switzerland, 3 studies (3%), contributed importantly, supported by collaborative networks and regional funding. Smaller contributions from Turkey (3), Brazil (2), and Chile (2), while single articles from countries such as Japan and Spain highlight a broad geographic reach. The VOSviewer coauthorship network highlights dense collaborations within Europe and Asia, with the United States fostering global partnerships. These patterns illustrate how regional proximity currently drives collaboration (Fig. 3).

### Affiliation-based collaboration patterns

**Figure 4:**
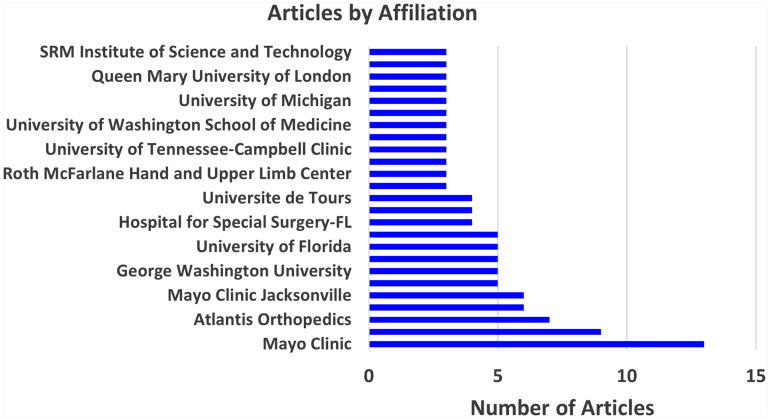
Artificial intelligence studies in shoulder and elbow per affiliation.

The analysis revealed 260 total affiliations that contributed to the AI research in shoulder and elbow surgery, including 93 unique affiliations based on first-author records. The Mayo Clinic had 13 publications, followed by the Hospital for Special Surgery (9) and Atlantis Orthopedics (7). Affiliations such as Exactech (6) and the Bordeaux-Merignac Sport Clinic (5). Coauthorship analysis identified distinct clusters reflecting thematic or geographic alignments, such as strong North American and trans-Atlantic collaboration (Fig. 4).

**Figure 5:**
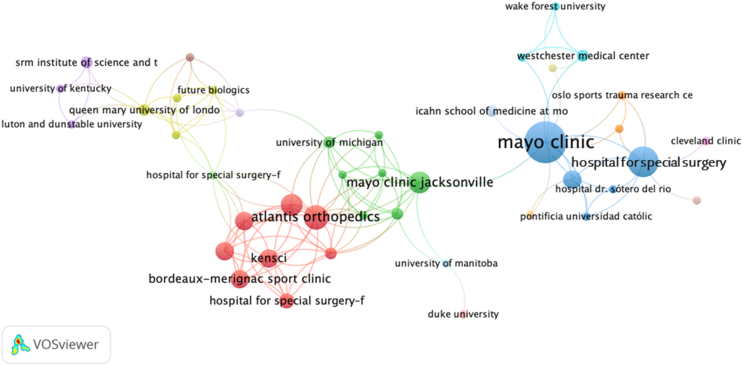
Affiliation-based collaboration network in artificial intelligence research for shoulder and elbow surgery, showing prominent clusters led by core groups.

The qualitative analysis coauthorship network visualization identified key affiliations forming at least 4 distinct clusters (Fig. 5)

1. Blue cluster (North America): This includes Mayo Clinic, Hospital for Special Surgery, Cleveland Clinic, and Westchester Medical Center, suggesting strong U.S.-based collaboration networks among these affiliations.
2. Red cluster (North America and Europe): Features Atlantis Orthopedics, Hospital for Special Surgery Florida, and Bordeaux-Mérignac Sport Clinic.
3. Green cluster (North America): University of Michigan, Mayo Clinic Jacksonville, and University of Manitoba.
4. Purple cluster (Global): Affiliations like Queen Mary University (UK), SRM Institute (India), and U.S. universities.

North American affiliations dominate collaboration density with notable cross-regional partnerships involving Europe and South America.

### Affiliation-based collaboration network

The coauthorship network included 260 affiliations connected by 1,010 collaborative links. The mean degree centrality was 5.2, indicating that, on average, each institution collaborated with 5 others. The network density was 0.03, showing that only 3 percent of all possible connections were realized. The modularity score was 0.81, indicating a strong community structure and the presence of tightly connected clusters with limited overlap between them (Table IV).

**Table IV:** Collaboration network metrics

Metric	Value	Description/Interpretation
Number of affiliations	260	Individual affiliations identified in the dataset
Number of collaborations	1,010	Total coauthorship links between distinct affiliations
Mean degree centrality	5.2	Average number of institutional connections per affiliation
Network density	0.03	Proportion of observed to possible links; indicates overall connectivity
Modularity (Louvain method)	0.81	Strength of community structure; higher values reflect distinct clusters

### Regression analysis on affiliation-based collaboration

**Figure 6:**
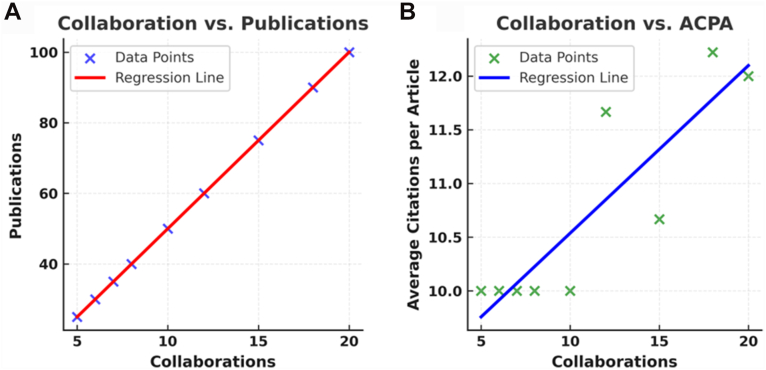
(**A**) Relationship between the number of collaborations and the total number of publications, showing a strong positive correlation with a fitted regression line; (**B**) Relationship between the number of collaborations and the average citations per article (ACPA), illustrating an increasing trend in citation impact with greater collaboration, as indicated by the regression line.

Higher collaboration levels were associated with increased publication output and greater per-article citation impact. In the first model, collaboration based on affiliation strongly predicted publication output with a coefficient of determination (R^2^) of 1.0. This indicates that 100% of the variance in publication output can be explained by the number of collaborations. Each additional collaboration was associated with an increase of 5 publications, demonstrating the direct impact of partnerships on research productivity (*P* < .001) (Fig. 6*A*, Table V).

**Table V:** Regression models results

Model	R Squared	Regression coefficient	*P* value	Interpretation
Model 1: collaborations → number of publications	1.00	+5 publications per additional collaborator	<.001	Stronger collaboration strongly predicts publication output
Model 2: collaborations → average citations	0.77	+0.2 citations per additional collaborator	.002	Greater collaboration is associated with higher citation impact

In the second model, collaboration levels were significantly associated with ACPA with an R^2^ of 0.77. This demonstrates that 77% of the variance in the AC was explained by the number of collaborations. Each additional collaboration increased the AC by 0.2 citations, suggesting a positive influence of collaboration on the impact and visibility of individual research articles (*P* = .002) (Fig. 6*B*, Table V).

## Author contributions

**Figure 7:**
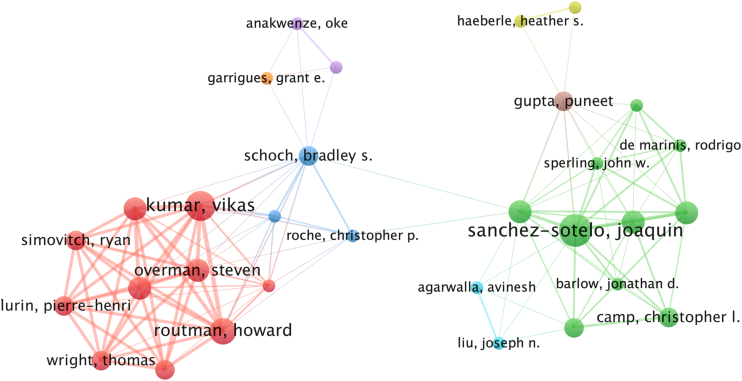
Author collaboration network in artificial intelligence research for shoulder and elbow surgery.

The analysis revealed a robust network of collaboration among researchers, with 623 NCA in the dataset, reflecting an important scholarly engagement. The collaboration index or the average number of authors per article was 5.2. The coauthorship network revealed Kumar and Sanchez-Sotelo Joaquin as pivotal researchers anchoring 2 distinct clusters with strong intracluster ties (Fig. 7).

### Keyword analysis

**Figure 8:**
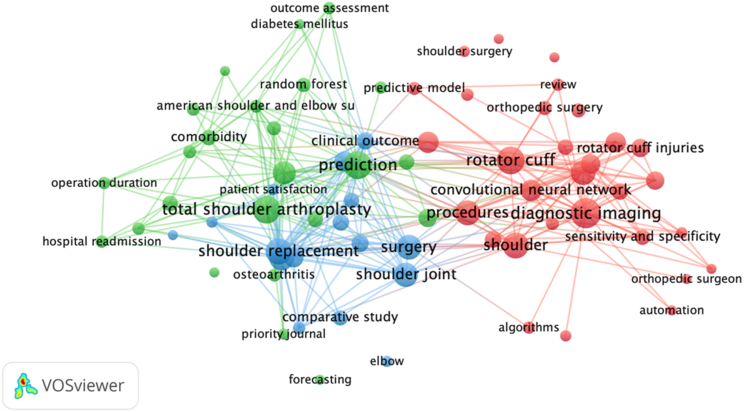
Keyword co-occurrence network in artificial intelligence research for shoulder and elbow surgery, illustrating major themes such as diagnostic imaging, rotator cuff injuries, total shoulder arthroplasty, and predictive modeling.

Co-occurrence network visualization organizes key research topics into thematic clusters, revealing their interconnections (Fig. 8). The green cluster seems to emphasize clinical outcomes and operational metrics in shoulder arthroplasty, including patient satisfaction and hospital readmission. The red cluster focuses on rotator cuff injuries and diagnostic imaging advancements such as imaging techniques and algorithms. The blue cluster bridges concepts between arthroplasty and diagnostic methods, highlighting broader topics, such as shoulder replacement, clinical outcomes, and surgery. These clusters provided a simplified overview of the current state of AI research in shoulder and elbow studies.

## Discussion

This study found the key role that affiliation-based collaboration plays in driving research productivity, especially in emerging topics. Collaborative cores, such as the Mayo Clinic and South Korean groups, demonstrate the strength of global and regional partnerships. However, the observed network density of 0.03, indicating that only 3% of all possible connections were realized, suggests that collaborative activity remains limited, with the vast majority of potential institutional partnerships unrealized. Similar patterns have been observed in other fields, where early-stage collaboration networks often show low connectivity due to geographic and institutional barriers.^11^ Although 1,010 collaborative links were identified among 260 affiliations, they are distributed unevenly, reflecting that many groups operate in relative isolation.

Furthermore, the dominance of countries such as the United States in research output contrasts with meaningful but underrepresented contributions from South Korea, China, and India. To reduce this disparity, capacity building in low-output regions can be achieved through international training programs, collaborative funding, and open data initiatives. Expanding networks, particularly interdisciplinary and cross-regional ties, could unlock greater innovation and enhance global research equity. These efforts will enhance global equity in research opportunities and outcomes.

The high modularity score was 0.81, reflects a network composed of distinct, tightly knit communities, likely shaped by geographic or thematic proximity. This aligns with previous studies, which emphasize that high modularity often corresponds to tightly knit clusters driven by regional or disciplinary priorities.^42^ Increasing cross-cluster collaboration may improve knowledge flow and support more globally integrated innovation in AI applications for shoulder and elbow surgery.

AI is becoming a game-changer in shoulder and elbow surgery, offering exciting possibilities for improving surgical planning and personalized patient care.^1^^,^^3^^,^^8^^,^^24^^,^^32^^,^^40^^,^^48^^,^^49^^,^^54^^,^^57^ It can automate complex analyses such as imaging diagnostics and predictive modeling, helping to enhance clinical outcomes.^2^^,^^6^^,^^9^^,^^10^^,^^14^^,^^21^^,^^22^^,^^35^^,^^37^, ^38^, ^39^^,^^41^^,^^46^^,^^51^^,^^53^^,^^63^^,^^64^ However, achieving the full potential of AI may require stronger and widespread collaboration. By fostering interdisciplinary and international partnerships, institutions can bring together diverse expertise to solve complex challenges and ensure that AI solutions address the most critical healthcare needs.^13^^,^^31^^,^^58^

The exponential rise in shoulder and elbow AI-related publications, particularly since 2020, reflects a growing interest in leveraging it for diagnostic precision, surgical planning, and patient outcomes.^24^^,^^40^ By 2024, research output peaked with 49 studies, illustrating the expanding adoption of these computational tools. This growth parallels global advancements in AI applications across orthopedics and healthcare in general.^13^^,^^58^ However, effective AI implementation would require interdisciplinary expertise to integrate clinical knowledge with engineering and computer science.^31^

Another key finding of this study was the significant relationship between affiliation-based collaboration and research productivity and impact. The regression analysis examined 2 models, both of which demonstrated strong correlation. Collaboration was quantified using degree centrality, which measured how many distinct institutions each affiliation partnered with. Regression models demonstrated that higher degree centrality was strongly correlated with greater publication output (R^2^ = 1.0) and with higher AC (R^2^ = 0.77). These findings indicate that institutions embedded in broader collaborative networks are more likely to generate both larger volumes of research and studies with greater visibility.

The second regression model explored the influence of collaboration on ACPA. The analysis showed an R^2^ value of 0.77, with each additional institutional collaboration increasing the AC by 0.2 citations (*P* = .002). This result highlights how partnerships not only boost research quantity but also could enhance quality and visibility. Collaborative efforts may lead to higher-impact studies by integrating diverse expertise and addressing complex research questions.

These results suggest the importance of fostering institutional networks to drive both productivity and scholarly impact of research in this field. Therefore, key hubs at the center of coauthorship networks could play a pivotal role in driving research output, setting priorities, and mentoring emerging contributors. Leadership-driven initiatives such as collaborative research grants and global mentorship programs are essential for fostering innovation and equity in the field. These institutions can also provide a blueprint for sustained regional and global research.

Preliminary thematic analysis revealed distinct clusters in diagnostic imaging, rotator cuff injuries, and arthroplasty optimization. These focus areas align with AI's strengths in improving diagnostic accuracy and surgical planning. However, the limited interconnections between thematic clusters suggest opportunities for greater interdisciplinary integration.

### Limitations

This study has several limitations. First, although Scopus is one of the most comprehensive and widely used databases, it is possible that some relevant studies indexed in other sources, such as Google Scholar, IEEE Xplore, or PubMed, were not included in our analysis. Second, we defined collaboration based on co-authorship between researchers with different affiliations. However, this metric does not always capture the spirit of collaboration. For example, in cases where authors list separate institutional affiliations but all data and resources derive from a single practice site, such relationships may represent nominal rather than substantive collaboration. Furthermore, we recognize that changes in author affiliations, such as transitions between residency, fellowship, and practice, may have influenced our findings. Furthermore, industry-driven collaborations may differ in nature and impact from academic institutional collaborations. Future studies should explore alternative methods to distinguish genuine institutional collaborations from administrative or industry-driven coauthorship and should aim to incorporate funding data to analyze its impact.

Third, most studies in our analysis were published after 2020, which may limit the reliability of citation metrics due to the time required for citations to accumulate. While citation counts are a widely used measure of scholarly impact, they may not fully capture the influence of recent publications. Furthermore, the lack of research quality or impact beyond bibliometric measures highlights the need for further qualitative and impact-focused evaluations. Future analyses should consider alternative impact measures, such as Altmetric scores or downloads, to complement citation-based metrics. Finally, this study offers a snapshot of a rapidly evolving field, making findings quickly outdated. Network analysis, while useful, may overlook nuances in research collaborations, particularly in sparse networks.^67^ Another limitation relates to the nature of the included studies. A substantial proportion consisted of reviews, commentaries, or descriptive analyses without primary data. Thus, while bibliometric mapping captures the volume and collaborations in this field, it may over-represent conceptual contributions relative to evidence-based clinical implementation.

Based on our findings, we suggest promoting strategies to boost the advancement of AI in shoulder and elbow surgery research. Increasing international collaboration and promoting interdisciplinary integration can address sparse network density and thematic gaps. Building AI research capacity in underrepresented regions and establishing standardized protocols for data, validation, and reporting could enhance equity and methodological rigor. Given that the field is at an inflection point and the literature expands rapidly, this analysis should be viewed as an early baseline rather than a definitive map. Future updates will be required to capture evolving trends and collaborations.

## Conclusion

This bibliometric analysis provides a comprehensive overview of AI research in shoulder and elbow surgery, highlighting the role of collaboration in driving productivity and impact. While our findings underscore the potential of partnerships to advance the field, they also reveal opportunities to expand collaboration networks and address regional disparities. Strengthening partnerships could accelerate progress and promote global participation. Future research should focus on fostering interdisciplinary and international collaborations to enhance the clinical applicability of AI in orthopedic practice.

## Disclaimers:

Funding: No funding was disclosed by the authors.

Conflicts of interest: The authors, their immediate families, and any research foundations with which they are affiliated have not received any financial payments or other benefits from any commercial entity related to the subject of this article.
