# Supplementary material for: Artificial intelligence in shoulder and elbow surgery: a bibliometric analysis of affiliation-based collaboration patterns
Source: JSES Int. 2025 Oct 14;10(1):101386. doi: 10.1016/j.jseint.2025.08.017 (PMC12681790; doi:10.1016/j.jseint.2025.08.017)
Supplement: Supplemental File 1 [file mmc1.docx]

**Supplemental File 1.**

The objective of this search was to identify scholarly articles that discuss the application of artificial intelligence (AI) technologies in the context of shoulder and elbow surgery. The search strategy was meticulously crafted to include key terms from three domains: AI technology, anatomical focus on the shoulder and elbow, and surgical interventions.

**Detailed Search Queries:**

**1. AI Technology Keywords:**

- **Title Search Query:**

TITLE ("artificial intelligence" OR "machine learning" OR ai OR "deep learning" OR "neural networks" OR "computer vision" OR "data mining" OR "predictive analytics" OR "natural language processing" OR nlp OR "large language models" OR llm OR gpt OR bert OR "transformer models" OR "transformers" OR "RoBERTa" OR "T5" OR "XLNet")

**2. Anatomical and Surgical Keywords:**

- **Title-Abstract-Keywords Search Query:**

TITLE-ABS-KEY ("shoulder" OR "elbow" OR "humerus" OR "rotator cuff" OR "tennis elbow" OR "triceps" OR "biceps" OR "acromioclavicular" OR "coracoclavicular" OR "sternoclavicular" OR "olecranon" OR "ulnar collateral ligament" OR "ulnar nerve" OR "radial head" OR "coronoid" OR "olecranon fossa" OR "PLRI" OR "PMRI" OR "proximal radius")

**3. Combining Both Queries with Surgical Procedures:**

- **Final Combined Query:**

AND TITLE-ABS-KEY (surgery OR arthroplasty OR arthroscopy OR surgical)
